# Multienzyme cascade biotransformation of L-Tyrosine to Danshensu by engineered *Escherichia coli*

**DOI:** 10.1371/journal.pone.0361484

**Published:** 2026-10-23

**Authors:** Tianzhen Xiong, Shuqing Li, Xiaoya Li, Xinmeng Li, Mengdan Wu, Jinru Liu, Jinxiu Guo, Xinrui Li

**Affiliations:** 1 Henan Key Laboratory of Tea Plant Biology, College of Tea and Food Science, Xinyang Normal University, Xinyang, Henan, China; 2 Dabie Mountain Laboratory, Xinyang, Henan, China; 3 Henan International Joint Laboratory of Tea-oil tree Biology and High-Value Utilization, College of Tea and Food Science, Xinyang Normal University, Xinyang, Henan, China; Foshan University, CHINA

## Abstract

Danshensu (DSS), a water-soluble bioactive compound with significant antioxidant, anti-inflammatory, cardioprotective and neuroprotective properties, is widely utilized in cardiovascular and cerebrovascular disease. In this study, we established a multi-enzyme cascade system in engineered *Escherichia coli* to synthesize DSS from L-Tyrosine. The initial co-expression of 4-hydroxyphenylacetate 3-monooxygenase (HpaBC), L-amino acid deaminase (aadL), glucose dehydrogenase (gdh) and D-lactate dehydrogenase (ldhD) yielded 5.1 mM DSS. The production of DSS increased to 6.53 mM through optimizing plasmid copy number. After optimizing the conditions, the yield of DSS reached 8.66 mM from strain 6. Finally, whole cell biotransformation using strain 6 in bioreactor yielded 19.47 mM DSS within 32h. These findings provide valuable insights for the scalable biosynthesis of pharmaceutically important compound of DSS from naturally available substrates of L-Tyrosine.

## 1 Introduction

Danshensu (DSS) has emerged as promising therapeutic agent owing to multifaceted pharmacological effects including antioxidant capacities [1], anti-inflammatory [2], cardioprotective [3] and enhancing cardiac performance [4]. In addition to being directly used as medicine, DSS can also serve as key precursor for synthesis of valuable derivatives. Danshensu Bingpian Zhi (DBZ) was synthesized using DSS and borneol as substrates and can enhance the therapeutic effect of cardiovascular and cerebrovascular diseases compared to DSS [5]. Isopropyl dihydroxyl hydroxy propionate (IDHP) was synthesized using DSS and isopropyl alcohol as substrates and can enhance the protective effect on the heart muscle compared to DSS [6].

Many methods have been used for production of DSS. Current industrial production of DSS primarily relies on extraction from *Salvia miltiorrhiza* roots [7]. However, the extraction of DSS from *Salvia miltiorrhiza* faces significant limitations. The resources of *Salvia miltiorrhiza* are limited, a large amount of organic solvents are used, many by-products are produced and the purification of DSS is difficult [8]. Meanwhile, conventional chemical synthesis routes starting from benzaldehyde is facing complex multi-step processes and inadequate stereochemical control [9]. In recent decades, synthetic biology technology has rapidly developed and has been used to produce large number of high-value natural products [10,11]. Biotechnological methods for the sustainable production of DSS conform to modern green chemistry principles.

Previous studies have explored the catalytic production of DSS by combining biological and chemical methods. Rao et al. developed a chemo-enzymatic route where L-DOPA was first chemically converted to 3,4-dihydroxyphenylpyruvic acid (DOPP), followed by enzymatic reduction to DSS using dual dehydrogenase system [12]. Metabolic engineering can produce DSS by specifically modifying the metabolic networks of microorganisms or plants. The hydroxylase complexes and D-lactate dehydrogenase were introduced into *Escherichia coli* to reconstruct DSS synthesis pathway; By knocking out the competitive pathway, enhancing the supply of precursor tyrosine, and combining with the regulation of the fermentation process, the yield of DSS was significantly increased. Finally, the yield of DSS reached 7.1g/L [13]. The engineered strain constructed by multi-enzyme cascade using cheap substrates as raw materials can also be used to produce DSS. Xiong et al. constructed engineered strain by heterologous expression of L-amino acid deaminase (aadL), D-lactate dehydrogenase (ldhD), glucose dehydrogenase (gdh) or tyrosine aminotransferase (tyrB), glutamate dehydrogenase (cdgdh), aromatic lactate dehydrogenase to produce DSS using L-DOPA as the substrate [14,15]. Subsequently, Xiong et al. heterologous expressed Tyrosin-phenol lyase (TPL), aadL, ldhD and gdh to construct engineered strain for production of DSS using catechol and pyruvic acid as substrates [16]. Zhang constructed a cascade reaction system by co-expressing 4-hydroxyphenylacetate 3-hydroxylase (HpaBC), aadL, D-lactate dehydrogenase and formate dehydrogenase using L-Tyrosine as the substrate to produce DSS [17].

In this study, we utilized the readily available natural compound L-Tyrosine as the substrate and constructed engineered strain that incorporates HpaBC, aadL, ldhD and gdh to product DSS (Fig 1). The production of DSS was boosted by optimizing culture conditions and fine-tuning pathway genes expression. In comparison with conventional chemical processes, whole-cell biotransformation offers several distinct advantages. These include the capacity to occur under mild conditions (low temperatures and pressures), the absence of utilisation of hazardous chemicals and the elimination of requirement for costly, noxious heavy metal catalysts. In contrast to fermentation-based methods, whole-cell biotransformation has been shown to yield greater yields and to facilitate downstream processing.

**Fig 1:**
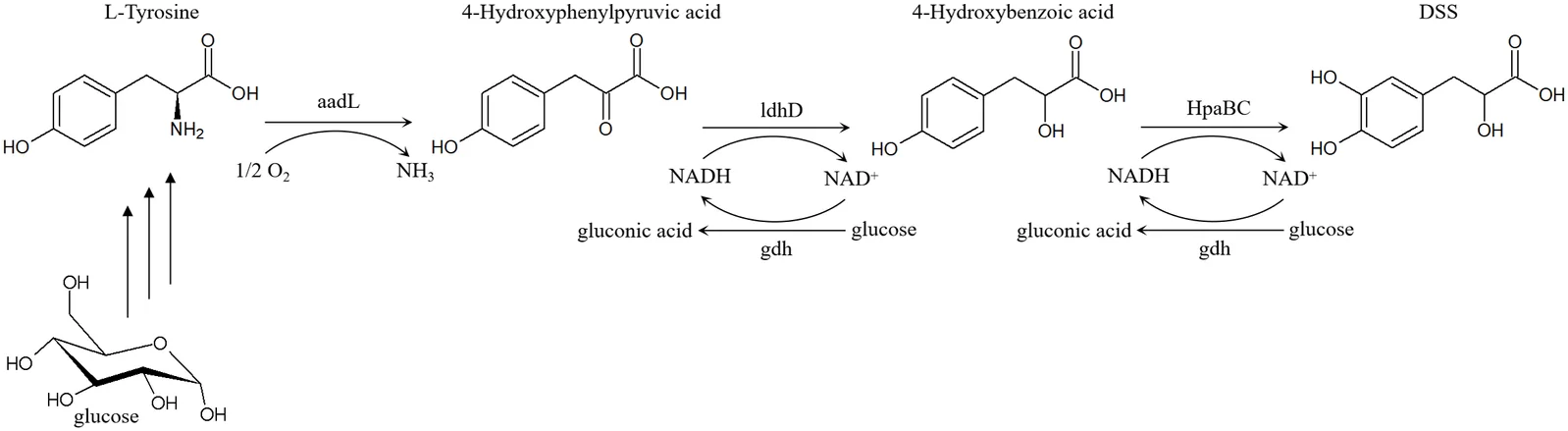
The proposed pathway for biosynthesis of DSS. aadL: L-amino acid deaminase, gdh: glucose dehydrogenase, ldhD: D-lactate dehydrogenase, HpaBC: 4-hydroxyphenylacetate 3-monooxygenase.

## 2 Materials and methods

### 2.1 Construction of plasmids and strains

The genes of *HpaBC* (*HpaB* GenBank ID: ARI00017, *HpaC* GenBank ID: ARI00016) were amplified from *Escherichia coli* BL21(DE3). The mutant (23F9-M4) was derivatives from *HpaB* with mutations at T15P, S210F, A211K, Q212F and D284E [18,19] and was used in the construction of engineered strains. *aadL* (GenBank ID: MH192985) [20], *ldhD* (GenBank ID: MG653257) [21] and *gdh* (GenBank ID: MH192986) [22] were obtained from previous studies. All successfully constructed plasmids were verified by sequencing from Tongyong (Anhui, China). Table S1 in S1 File lists primers, Table S2 in S1 File lists plasmids and strains.

### 2.2 RT-qPCR analysis

The relative abundance of messenger RNAs was determined using real-time quantitative PCR (RT-qPCR). Total RNA was extracted using RNA extraction kit (TaKaRa, Dalian, China) from the log-phase culture cells. Complementary DNA and qPCR were performed in one step on a CFX Opus 96 Detection System (bio-rad, America) using the One Step TB Green PrimeScript RT-PCR kit from TaKaRa (Dalian, China). The primers used for the *HpaBC*, *aadL*, *ldhD* and *gdh* genes in the RT-PCR experiments listed in Table S1 in S1 File. The 16S ribosomal RNA is accepted as a stably expressed house-keeping gene and was used as the internal reference gene for relative expression analysis [23,24]. Each amplification was performed three times.

### 2.3 The expression of heterologous genes

The constituted *Escherichia coli* was transferred to 3mL TB medium and then cultured for 12 h at 37°C, 220 rpm. Subsequently, 50 μL culture medium was inoculated to 50 mL TB medium and the culture was continued at 37°C, 220 rpm until the OD_600_ value reached 0.9–1. Thereafter, isopropyl-β-D-thiogalactoside (IPTG) was incorporated into the culture medium at final concentration of 0.5 mM and then the culture was subjected to further incubation at temperature of 25°C and at speed of 220 rpm for 12 h. The cultured cells were collected by means of centrifugation (4°C, 8000 rpm), and then the centrifugate precipitate was washed twice with 20 mM phosphate buffer and resuspended in 20 mM phosphate buffer.

### 2.4 The production of DSS using whole-cell biotransformation

The reactions were carried out in 50 mL reaction mixtures within 250 mL flasks. The reaction system was composed of 50 mM glucose, 25 mM L-Tyrosine and 50 g/L wet cells. The reaction conditions were established at temperature of 30°C, pH of 7 with rotation speed of 150 rpm and reaction time of 6 h. Subsequently, the reaction mixture was subjected to boiling for 2 min to inactivate the enzymes. Following this, centrifugation was employed at 8000 rpm for 2 min with the aim of removing the bacterial cells from the mixture. All biological experiments were carried out in three independent replicates. The analysis was conducted using high-performance liquid chromatography (HPLC).

### 2.5 Optimization of protein expression conditions

Strain 6 was inoculated into flask with TB medium. The flask was filled with 50 mL reaction system. To determine the optimal induction conditions for recombinant protein expression, a series of single-factor experiments were conducted. To evaluate the effect of cell density, *E. coli* cultures were grown to densities at OD_600_ of 0.32, 0.63, 0.93, 1.21, 1.53, and 1.91, respectively (Following induction with 0.5 mM IPTG at 25°C for 12 h). To evaluate the effect of final IPTG concentration, IPTG at final concentrations was 0.1 mM, 0.25 mM, 0.5 mM, 0.75 mM, 1.03 mM, 1.22 mM, respectively (Following induction with OD_600_ of 0.93 at 25 °C for 12 h). The effect of induction temperature was assessed by culturing the recombinant strain at OD_600_ of 0.93 and inducing with 0.5 mM IPTG. The cultures were then incubated at temperatures of 15°C, 20°C, 25°C, 30°C, 35°C, 40°C for 12 h. To identify the optimal induction time, cultures were induced at OD_600_ of 0.93 with 0.5 mM IPTG at 25°C and harvested at 6 h, 9 h, 12 h, 15 h, 18 h, 21 h, respectively. All biological experiments were carried out in three independent replicates.

### 2.6 Optimization of whole-cell biotransformation conditions

The reaction system comprised wet cells weight of 50 g/L, in addition to 50 mM glucose and 25 mM L-Tyrosine. The pH value was optimised through careful control of reaction temperature (35°C) and reaction time (6 h). To ensure precise pH adjustment, 20 mM phosphate buffer and Tris-HCl buffer were utilized with pH values ranging from 6 to 8.5. Following this adjustment, the content of DSS was measured under various pH values conditions. The temperature was optimised through control of the pH value (7.5) and the reaction time (6 h). The temperature was then adjusted to 20°C, 25°C, 30°C, 35°C, 40°C and 45°C, respectively. The content of DSS was measured under different temperature conditions. For optimization of wet cells weight, the conditions were 35°C, pH 7.5 and wet cells weight between 20 and 70 g/L. All reactions were stopped by boiling for 1 min and then centrifugation at 10000 rpm for 5 min. All supernatants were retained for HPLC.

### 2.7 Producing DSS in bioreactor

The strain 6 and strain 7 were inoculated into TB medium and subsequently cultured at temperature of 35°C with shaking rate of 220 rpm, duration of 12 h. Thereafter, a seed culture of 50 mL was introduced into 3 L TB medium within 5 L bioreactor. The temperature of the experiment was calibrated to a target level of 35°C and the initial ventilation rate was configured at 5 L/min. The dissolved oxygen level was sustained at 20% by adjusting the stirring speed within the range of 100 rpm to 500 rpm. The pH level of the medium was modified using hydrochloric acid and sodium hydroxide. When the concentration of engineering strain (OD_600_) was 0.93, IPTG was introduced to 3L TB medium at 0.5 mM (final concentration). Thereafter, the culture temperature underwent an adjustment to 25°C with the culture being maintained for a period of 12 h. Following the process of cultivation, wet cells were extracted by centrifugation (8000 rpm, 4°C, 5 min).

The volume of biotransformation system is 1.5 L within 5 L bioreactor and the biotransformation system composed of 50 mM glucose, 25 mM L-Tyrosine and 50 g/L wet cells. The stirring rate and temperature were constant at 150 rpm and 35°C, respectively. The pH value was stabilised through the addition of hydrochloric acid and sodium hydroxide ensuring a consistent level of 7.5. Samples were obtained at hourly intervals. Following the transformation, the bacterial cells were removed by means of centrifugation at 8000 rpm, 4°C for 5 min. Thereafter, the sample was subjected to filtration via 0.22 μm microporous membrane. The composition of each sample was ascertained by HPLC. All biological experiments were carried out in three independent replicates.

### 2.8 Analysis methods

HPLC (Agilent Technologies, California, U.S.A) and Nuovasil column (4.6 × 250 mm, 5 μm) was utilised for separation of the substances present within the samples. The analytical method was based on previous research [14].

## 3 Results and discussion

### 3.1 Select appropriate heterologous genes for cascade reaction to produce DSS

Previous researches have enhanced the activity of HpaB through random mutations and high-throughput screening methods [18,19]. The final positive mutant (23F9-M4) was selected. The aadL has been demonstrated to be capable of efficiently converting L-DOPA into 3,4-dihydroxyphenylpyruvic acid [20]. ldhD has been demonstrated to reduce 3,4-dihydroxyphenylpyruvic acid to DSS [21]. gdh has been demonstrated to be capable of efficiently converting NAD^+^ to NADH, thereby achieving cofactor regeneration [22]. Consequently, the selection of genes were undertaken for constructing engineered strains.

Due to HpaBC and ldhD exhibit promiscuous substrate specificity, DSS can be synthesized through two pathways (Fig 2A) [17]. In pathway 1, ldhD catalyzed the conversion of 4-Hydroxyphenylpyruvic acid to 4-Phenyllactic acid, which was then hydroxylated to DSS by HpaBC. In pathway 2, HpaBC catalyzed the conversion of 4-Hydroxyphenylpyruvic acid to 3,4-Dihydroxyphenylpyruvic acid, which was then reduced to DSS by ldhD.

**Fig 2:**
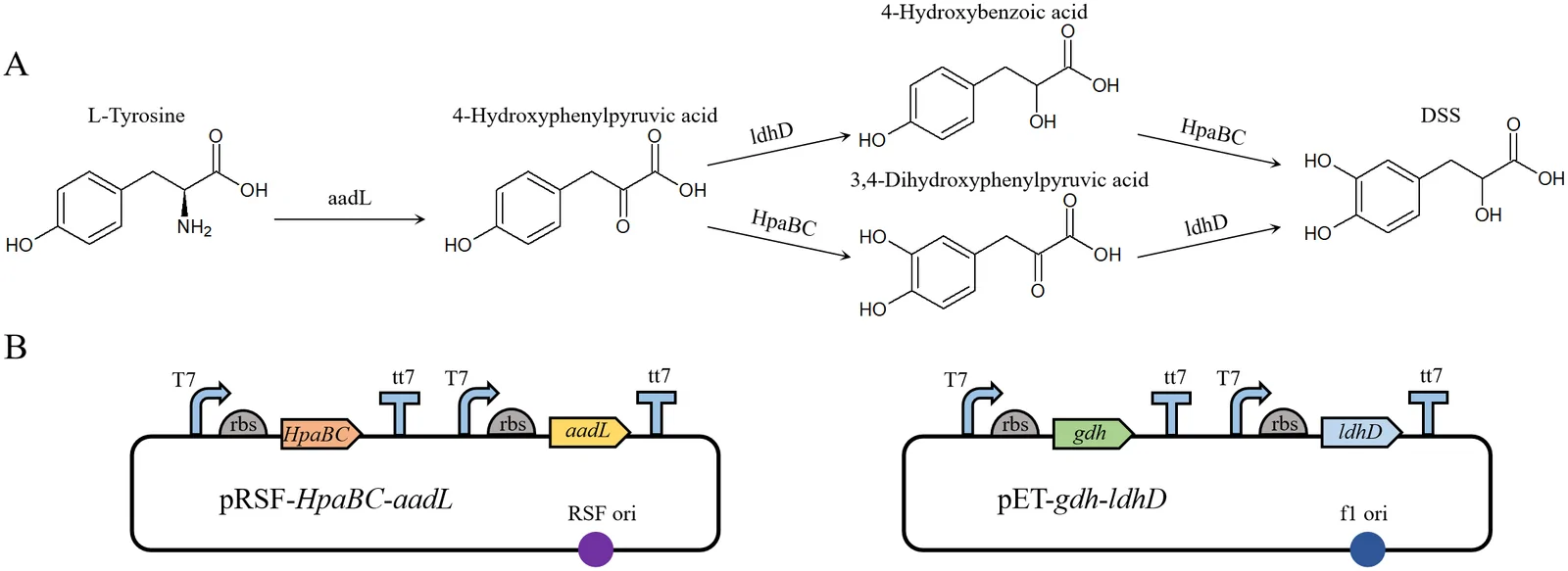
Target reaction scheme. (A) Construction of the pathway for DSS synthesis from L-Tyrosine. (B) Coexpression of the pathway genes in pRSFDuet-1 and pETDuet-1.

The strain 1 was achieved through utilisation of pRSFDuet-1 and pETDuet-1, which were employed to express *HpaBC*, *aadL*, *ldhD* and *gdh*. In this study, the medium-copy-number plasmid of pETDuet-1 was employed for expression of *ldhD* and *gdh*, while the high-copy-number plasmid of pRSFDuet-1 was employed for expression of *HpaBC* and *aadL*. We constructed plasmids of pRSF-*HpaBC*-*aadL* and pET-*gdh*-*ldhD* (Fig 2B) and then introduced these plasmids into *E. coli* BL21 (DE3) to construct strain 1 (Fig 3A). Whole-cell biotransformation was conducted using strain 1 with L-Tyrosine as substrate. After 6 h, 5.1 mM DSS was obtained. The reaction was analyzed using combination of liquid chromatography and mass spectrometry (Fig S1 in S1 File). The fragment distribution of reaction product spectrum and the full-wavelength scanning pattern are consistent with DSS standard [25].

**Fig 3:**
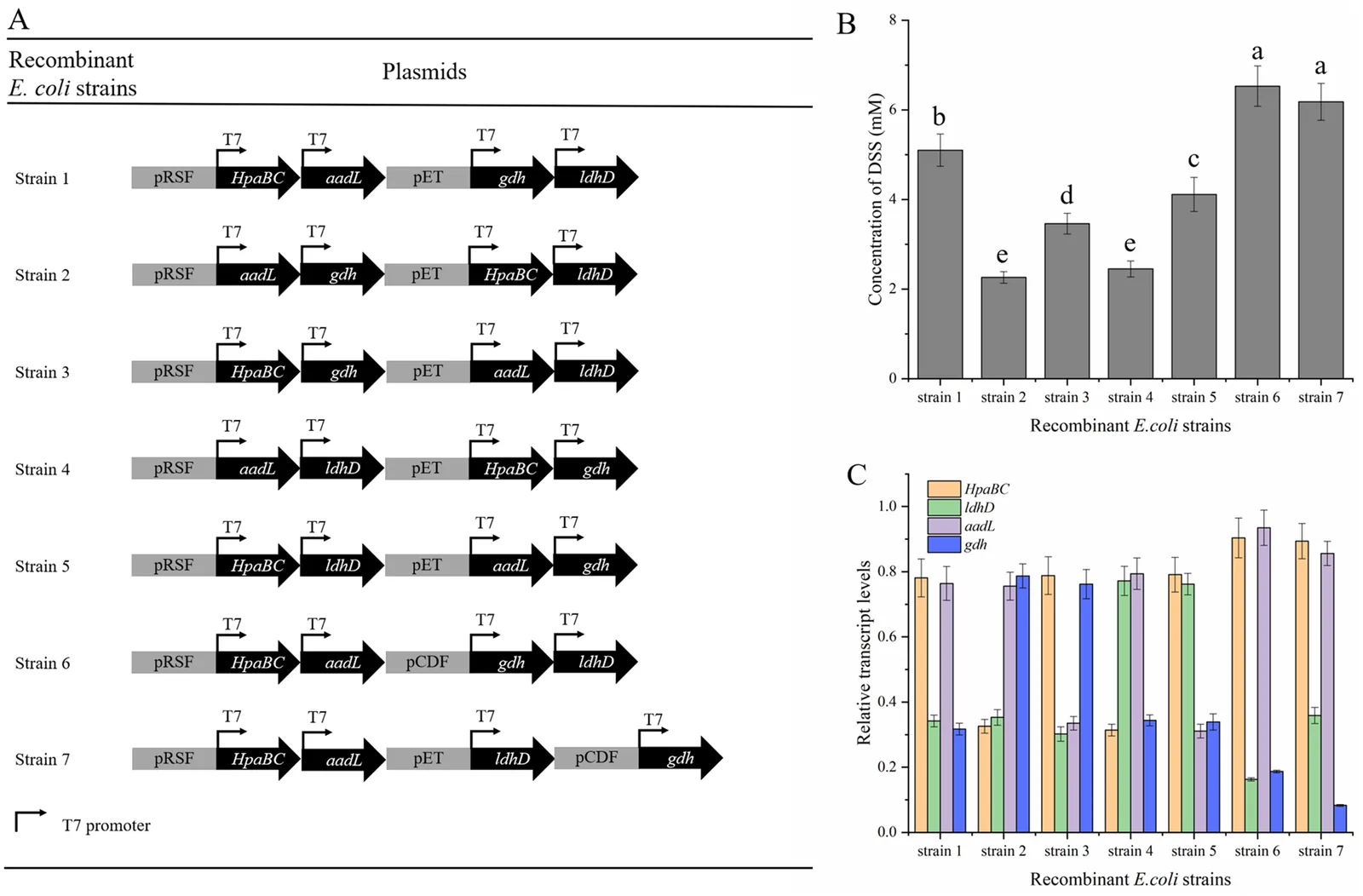
The process of whole cell biotransformation through the engineering of *Escherichia coli.* (A) The construction of composite plasmids for co-expression of *HpaBC*, *aadL*, *ldhD* and *gdh*. (B) The production of DSS from engineered strains. (C) The relative transcript levels of *HpaBC* (yellow box), *aadL* (green box), *gdh* (blue box) and *ldhD* (purple box) in various types of engineered strains. Values with different superscript letters are significantly different across columns (P < 0.05).

### 3.2 Enhance production of DSS through balanced pathway genes expression

Whole-cell biotransformation has emerged as robust technique for effective production of natural products [26,27]. In context of whole-cell biotransformation, primary objectives are to enhance the efficiency of biosynthetic pathways. A frequently employed stratagem to attain efficacious production of target compounds is to achieve an equilibrium in expression levels of pathway genes [28]. The enhancement of production capacity of engineered strains has been shown to be possible through the combination of plasmids with different copy numbers and target genes [29,30]. We employed plasmids with different copy numbers to express pathway genes. The expression level of genes within the seven strains of *E. coli* were each measured in reverse RT-qPCR assay and the representation of their comparative transcript levels is shown in Fig 3C.

A comparison was made between strain 1 and strain 2. In strain 2, *HpaBC* was expressed via the medium-copy-number plasmid of pETDuet-1, whereas in strain 1, *HpaBC* was expressed via the high-copy-number plasmid of pRSFDuet-1. The relative transcription level of *HpaBC* in strain 1 was found to be 2.4 times higher than strain 2 (Fig 3C). HPLC analysis indicated a significant disparity in production of DSS between strain 2 (2.26 mM) and strain 1 (5.1 mM) as illustrated in Fig 3B. This result indicates that the necessity of robust *HpaBC* induction for enhancement of DSS production in strain 2. The strain 3 (pET-*aadL*-*ldhD*, pRSF-*HpaBC*-*gdh*) was constructed to investigate the impact of *aadL* on production of DSS. In strain 3, high-copy-number plasmid of pRSFDuet-1 was utilised for expression of *HpaBC* and *gdh*, while medium-copy-number plasmid of pETDuet-1 was employed for *aadL* and *ldhD*. HPLC analysis indicated that the yield of DSS in strain 3 (3.46 mM) was lower than strain 1. The strain 4 (pET-*HpaBC*-*gdh*, pRSF-*aadL*-*ldhD*) was constructed. In strain 4, high-copy-number plasmid of pRSFDuet-1 was utilised for expression of *aadL*, *ldhD*, while medium-copy-number plasmid of pETDuet-1 was employed for *HpaBC* and *gdh*. HPLC analysis indicated that DSS in strain 4 (2.45 mM) diminished considerably in comparison with strain 1, accounting for 48.1% level in strain 1. To investigate the impact of *ldhD* on production of DSS, the strain 5 (pET-*aadL*-*gdh*, pRSF-*HpaBC*-*ldhD*) was constructed. In strain 5, high-copy-number plasmid of pRSFDuet-1 was utilised for expression of *HpaBC* and *ldhD*, while medium-copy-number plasmid of pETDuet-1 was employed for *aadL* and *gdh*. Furthermore, the relative transcription level of *aadL* in strain 5 has been shown to exceed strain 1 (Fig 3C). HPLC analysis revealed that the production of DSS in strain 5 (4.11 mM) exhibited a slight decrease in comparison to strain 1.

The strain 6 (pCDF-*gdh*-*ldhD*, pRSF-*HpaBC*-*aadL*) was then constructed. The expression of *HpaBC* and *aadL* was achieved by utilising high-copy-number plasmid of pRSFDuet-1, *gdh* and *ldhD* was achieved by utilising low-copy-number plasmid of pCDFDuet-1. HPLC analysis demonstrated that the synthesis yield of DSS in strain 6 (6.53 mM) exhibited substantial increase compared with strain 1, attaining 1.3-fold that of strain 1. Finally, strain 7 was subjected to further analysis. Strain 7 was found to express *HpaBC*, *aadL* in pRSFDuet-1 and *ldhD* in pETDuet-1 and *gdh* in pCDFDuet-1. HPLC analysis revealed the production levels of DSS in strain 7 (6.18 mM) to be lower than strain 6. In summary, the strain 6 demonstrated the capacity to generate the highest levels of DSS in comparison to other strains, exhibiting 28.1% increase in DSS production relative to strain 1.

The employment of compatible plasmids to regulate the expression of pathway enzymes has been demonstrated to enhance the yield of DSS. The engineered *Escherichia coli* exhibited variable degrees of DSS production capacity as demonstrated in Fig 3B. The HpaBC is characterised as exhibiting comparatively lower levels of activity in comparison to aadL, ldhD and gdh. The hydroxylation reaction from HpaBC may be the rate-limiting step in the generation process of DSS [31]. The *HpaBC* was expressed in pRSFDuet-1 (high copy number), which was found to be effective method for increasing the yield of DSS (strain 6, strain 7). gdh is extensively utilised in NADH cofactor regeneration [32,33]. The *gdh* is expressed on low-copy-number plasmid of pCDFDuet-1 (strain 6), leading to increase DSS production in comparison to other strains. The relatively low levels of *gdh* expression may be sufficient to meet the demand for cofactor regeneration [34].

### 3.3 Optimize the culture conditions of bacteria

To establish the reaction to produce DSS from L-Tyrosine, the production of DSS was first improved by optimizing various parameters of induction condition. Through these optimization processes, the results showed that the optimal induction OD_600_ value is 0.93 (Fig 4A), the optimal induction IPTG final concentration is 0.5 (Fig 4B), the optimal induction temperature is 25°C (Fig 4C) and the optimal induction time is 12 h (Fig 4D). The genes were expressed under the control of inducible T7 promoter in recombinant *Escherichia coli*. Inappropriate induction OD_600_, inducer IPTG concentration, and induction time will be toxic to cells and affect heterologous protein synthesis [35].

**Fig 4:**
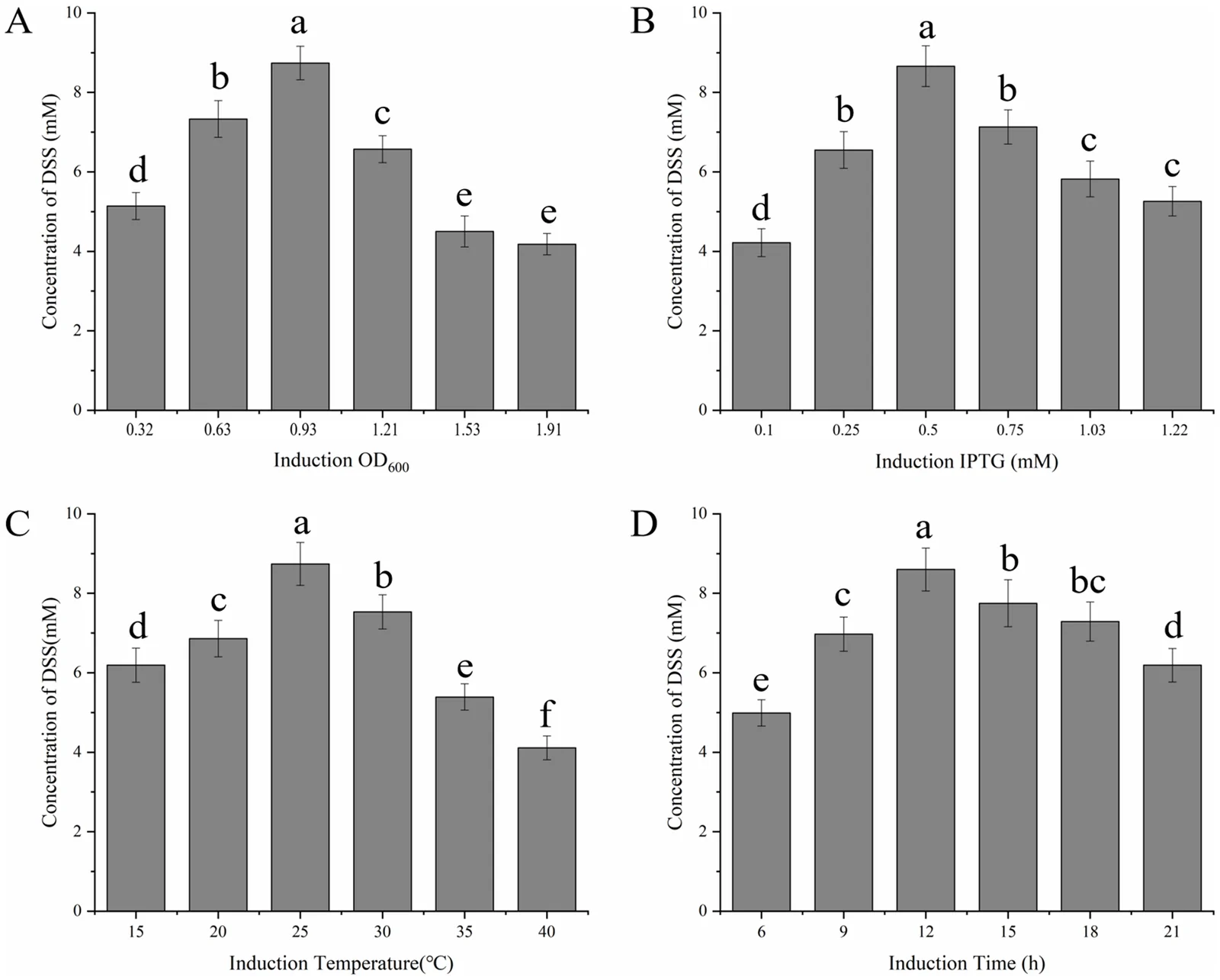
The impact of inducing conditions on production of DSS by strain 6, (A) induction OD_600_, (B) induction IPTG, (C) induction temperature, (D) induction time. Values with different superscript letters are significantly different across columns (P < 0.05).

Subsequently, the reaction conditions of whole cell biotransformation were systematically studied. In order to optimize temperature, the yield of DSS exhibited marked tendency to increase within range of 20°C to 35°C and to decrease within range of 35°C to 45°C. The most effective temperature for the process was found to be 35°C, which resulted in greatest yield of DSS. The final concentration of DSS was 8.66 mM (Fig 5A). In order to optimize pH, an increase in production of DSS was observed as the pH fluctuated between 6 and 7.5. As the pH level varied from 7.5 to 8.5, a concomittant decline in production of DSS was observed. The most effective pH was determined to be 7.5, which consequently resulted in the maximum yield of DSS (Fig 5B). When investigating the optimal wet cells weight for production of DSS, the production of DSS can be increased by increasing the amount of wet cells weight. The yield of DSS was not increased linearly (Fig 5C). The maximum-specific activity for DSS production obtained at a cell biomass of 50 g/L. The optimization was performed by changing one factor at a time. This method is difficult to evaluate interactions between IPTG concentration, induction temperature, induction time, pH, and cell concentration. Future studies will employ multivariate statistical models to systematically investigate these interactions and further refine the expression strategy.

**Fig 5:**
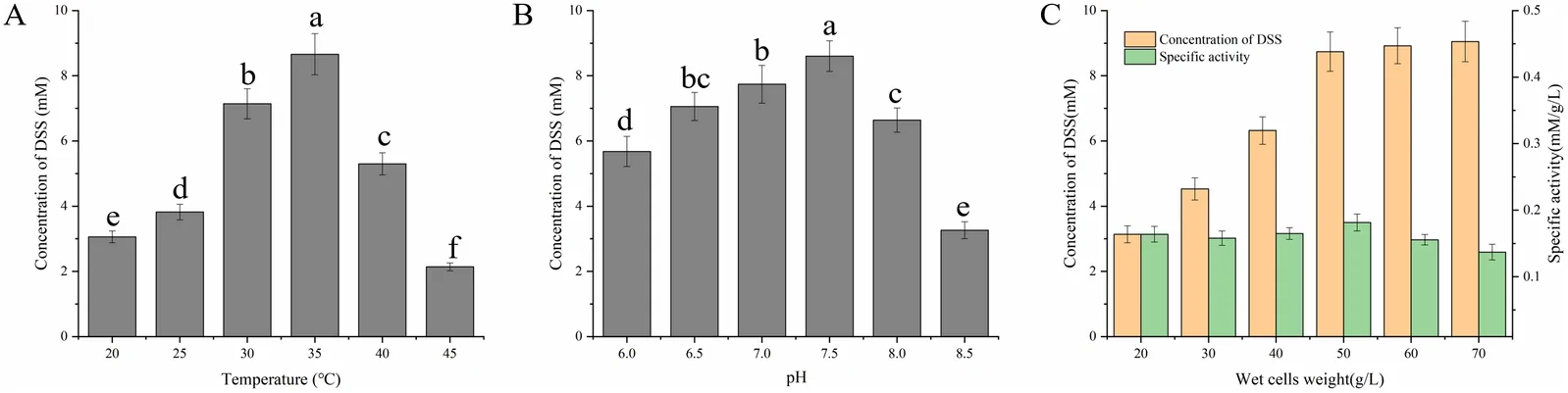
The impact of reaction conditions on production of DSS by strain 6, (A) temperature, (B) pH, (C) wet cells weight. Values with different superscript letters are significantly different across columns (P < 0.05).

### 3.4 Whole-cell biotransformation for production of DSS in bioreactor

The whole-cell biotransformation was carried out in bioreactor from strain 6 and strain 7. We constructed plasmids of pCDF-*gdh*-*ldhD* and pRSF-*HpaBC*-*aadL*, which were introduced into *E. coli* BL21 (DE3) to construct strain 6. Additionally, we also constructed plasmids of pET-*ldhD,* pCDF-*gdh* and pRSF-*HpaBC*-*aadL*, which were introduced into *E. coli* BL21 (DE3) to construct strain 7 (Fig 6A).

**Fig 6:**
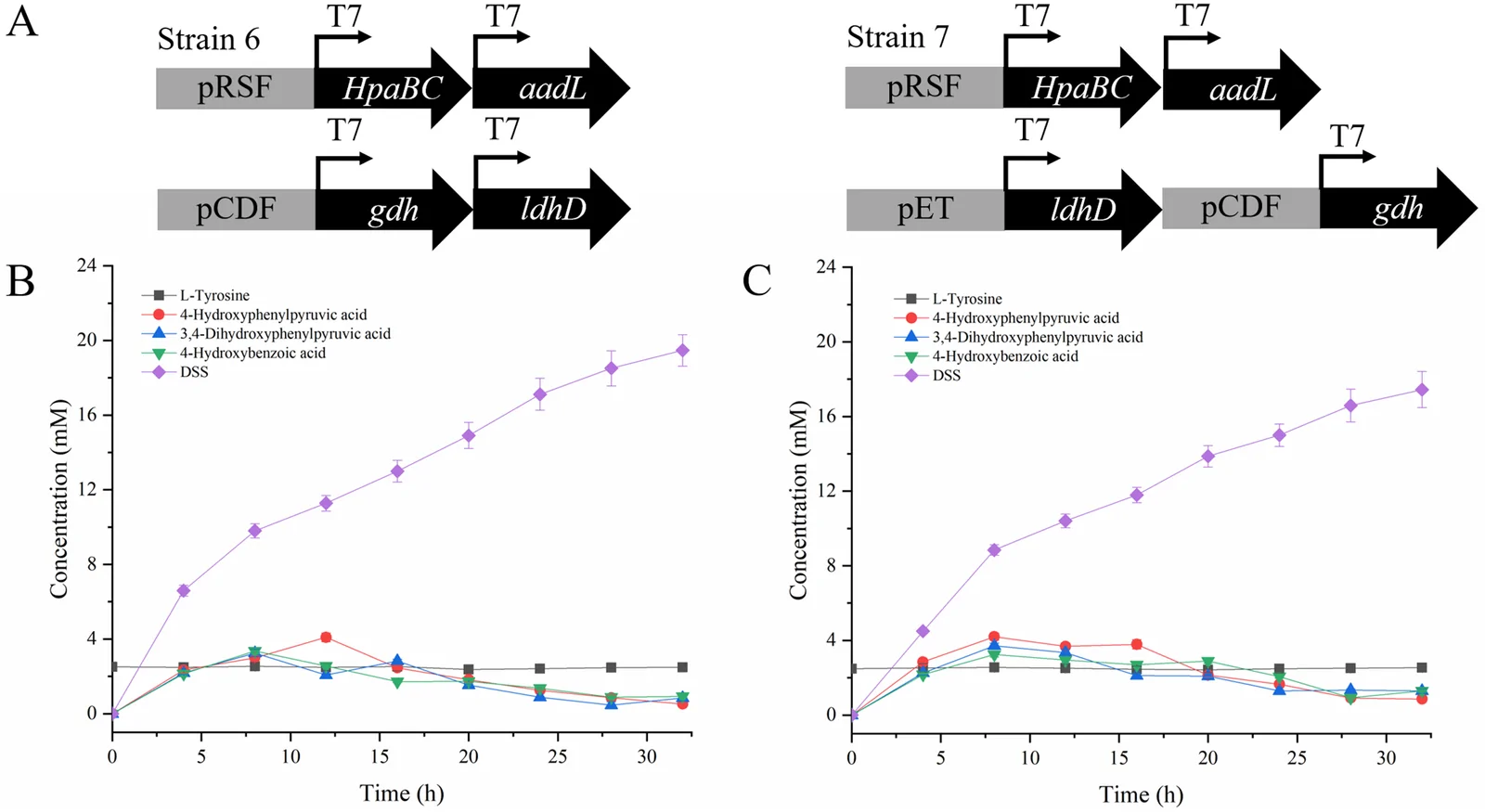
Scale-up the manufacturing of DSS in bioreactor, (A) The construction of composite plasmids from strain 6 and strain 7, (B) Time courses of product and intermediate production from strain 6, (C) Time courses of product and intermediate production from strain 7.

In strain 6, the 19.47 mM DSS was produced from 25 mM L-tyrosine. A few intermediates were generated (Fig 6B), suggesting that *HpaBC* and *aadL* might be the rate-limiting enzymes. In strain7, with the passing of time, we observed an increase yield of DSS, which coincided with decrease in content of L-Tyrosine. Finally, 25 mM L-Tyrosine produced 17.44 mM DSS (Fig 6C).

L-Tyrosine is a naturally available compound and easily obtainable and inexpensive. In most enzymatic production processes of DSS, glucose and L-DOPA are commonly used substrates. This study used L-Tyrosine as the substrate to synthesize DSS (Table 1) and provides a feasible strategy to convert the naturally available and easily obtainable L-Tyrosine into the high-value DSS. Zhang et al. used L-Tyrosine as the substrate to produce DSS. However, the reaction was not one-pot reaction and was carried out in three Step [17]. In this study, the multi-enzyme cascade reaction has greatly simplified the production process.

**Table 1:** Comparison of different methods for synthesizing DSS.

year	substrate	reaction time (h)	reaction scale (mL)	molar yield	concentration (g/L)	space-time yield (mg/L/h)	reference
2013	glucose	70	200	0.47 mol/mol	7.1	101.43	[13]
2017	glucose	60	2100	–	5.6	93.33	[36]
2018	3,4-dihydroxyphenylpyruvic	12	50	95.45%	2.25	187.5	[37]
2018	L-DOPA	12	20	95.3%	23.58	1965	[14]
2019	catechol	14	1500	–	10.96	782.86	[16]
2019	L-DOPA	18	50	95.12%	23.54	1307.78	[15]
2019	phenylpyruvate	12	1	84.66%	8.73	727.5	[38]
2022	L-DOPA	1	10	83.7%	6.64	6610	[39]
2023	L-DOPA	12	100	91.78%	82.6	6883.33	[40]
2025	glucose	48	3000	–	37.26	776.25	[41]
2025	L-Tyrosine	17	3	81.67%	16.17	951.18	[17]
2026	lignin	–	100	17.7 wt%	4.07	–	[42]
2026	L-Tyrosine	32	1500	77.88%	3.85	120.31	This work

## 4 Conclusions

In summary, the engineered *Escherichia coli* was achieved by co-expression of *HpaBC*, *aadL*, *ldhD*, *gdh* and has the capacity to convert L-Tyrosine to DSS. The synthesis of DSS was optimised through the enhancement of the combination of differing copy numbers plasmids and pathway genes. The present study has provided new ideas for production of DSS.

## Supporting information

S1 File Supplementary material for this article includes descriptions of the construction methods for the plasmids and the strains, UPLC−MS assay of DSS, Primers used in the work, Strains and plasmids used in this study. (DOCX)

S1 Data Minimal data set. (XLSX)

## References

[1] Guo C, Ma Z, Tao X, Gao K, Zhang W, Wen A, et al. Therapeutic time window of sodium of Danshensu on cerebral ischemia and its mechanism of inhibiting oxidative stress and ferroptosis through Nrf2 pathway. Brain Res Bull. 2025;227:111396. doi: 10.1016/j.brainresbull.2025.111396 40403934

[2] Huang Y, Lai Y, Chen L, Fu K, Shi D, Ma X, et al. Danshensu enhances autophagy and reduces inflammation by downregulating TNF-α to inhibit the NF-κB signaling pathway in ischemic flaps. Phytomedicine. 2025;137:156378. doi: 10.1016/j.phymed.2025.156378 39818119

[3] Lin C, Li W, Wen S, Ye D, Zhao Z, Huang L, et al. Cardioprotective effects of Salviae Miltiorrhizae Radix et Rhizoma on myocardial ischemia-reperfusion injury in animal models: A systematic review and meta-analysis. J Ethnopharmacol. 2026;368:121804. doi: 10.1016/j.jep.2026.121804 42097343

[4] Hu T, Wei G, Xi M, Yan J, Wu X, Wang Y, et al. Synergistic cardioprotective effects of Danshensu and hydroxysafflor yellow A against myocardial ischemia-reperfusion injury are mediated through the Akt/Nrf_2_/HO-1 pathway. Int J Mol Med. 2021;48(6). doi: 10.3892/ijmm.2021.5045 34633051 PMC8522956

[5] Wang J, Xu P, Xie X, Li J, Zhang J, Wang J, et al. DBZ (Danshensu Bingpian Zhi), a Novel Natural Compound Derivative, Attenuates Atherosclerosis in Apolipoprotein E-Deficient Mice. J Am Heart Assoc. 2017;6(10):e006297. doi: 10.1161/JAHA.117.006297 28971954 PMC5721843

[6] Yang Y, Du L, Zhao H, Zhao Y, Liao S, Zhang Z, et al. Danshensu Derivatives: A Series of Promising Drugs With Protective Effects Against Cardiocerebrovascular Diseases and Cancers. Med Res Rev. 2025;45(4):1163–83. doi: 10.1002/med.22102 40072268

[7] Peng R, Wu Q, Chen X, Ghosh R. Purification of danshensu from Salvia miltiorrhiza extract using graphene oxide-based composite adsorbent. Ind Eng Chem Res. 2017;56(31):8972–80. doi: 10.1021/acs.iecr.7b00661

[8] Chen J, Lee FS-C, Li L, Yang B, Wang X. Standardized extracts of Chinese medicinal herbs: Case study of Danshen (Salvia miltiorrhiza Bunge). Journal of Food and Drug Analysis. 2020;15(4). doi: 10.38212/2224-6614.2386

[9] Bai Y, Zhang Q, Jia P, Yang L, Sun Y, Nan Y, et al. Improved Process for Pilot-Scale Synthesis of Danshensu ((±)-DSS) and Its Enantiomer Derivatives. Org Process Res Dev. 2013;18(12):1667–73. doi: 10.1021/op4002593

[10] Ke J, Wan L, Chen M, Lv Y, Zhu Y, Zhang W, et al. Spatial engineering for biocatalytic cascade control through biomolecular compartmentalization. Biotechnol Adv. 2026;87:108786. doi: 10.1016/j.biotechadv.2025.108786 41475588

[11] Yang Q, Zhu FY, Zhang XJ, Cai X, Zhou JP, Liu ZQ. Driving in vivo multienzyme cascades forward: regulatory strategies for enhanced biocatalysis. ACS Synth Biol. 2026. doi: 10.1021/acssynbio.5c00951 41894637

[12] Rao NN, Roth HJ. Enzymatic synthesis of (S)-(-)-3-(3,4-dihydroxyphenyl)lactic acid. Arch Pharm. 1988;321(3):179–80. doi: 10.1021/acssynbio.5c00951 41894637

[13] Yao Y-F, Wang C-S, Qiao J, Zhao G-R. Metabolic engineering of Escherichia coli for production of salvianic acid A via an artificial biosynthetic pathway. Metab Eng. 2013;19:79–87. doi: 10.1016/j.ymben.2013.06.001 23774671

[14] Xiong T, Jiang J, Bai Y, Fan T-P, Zhao Y, Zheng X, et al. Biosynthesis of D-danshensu from L-DOPA using engineered Escherichia coli whole cells. Appl Microbiol Biotechnol. 2019;103(15):6097–105. doi: 10.1007/s00253-019-09947-0 31187210

[15] Xiong T, Jiang J, Bai Y, Fan TP, Zhao Y, Zheng X, et al. Redox self-sufficient biocatalyst system for conversion of 3,4-dihydroxyphenyl-l-alanine into (R)- or (S)-3,4-dihydroxyphenyllactic acid. J Ind Microbiol Biotechnol. 2019;46(8):1081–90. doi: 10.1007/s10295-019-02200-7 31201648

[16] Xiong T, Jia P, Jiang J, Bai Y, Fan T-P, Zheng X, et al. One-pot, three-step cascade synthesis of D-danshensu using engineered Escherichia coli whole cells. J Biotechnol. 2019;300:48–54. doi: 10.1016/j.jbiotec.2019.05.008 31125578

[17] Zhang M, Zhong J, Zhang Y, Wang W, Zhao W, Hu S, et al. Development of a Multienzyme Cascade for Salvianolic Acid A Synthesis from l-Tyrosine. ACS Omega. 2025;10(5):4792–800. doi: 10.1021/acsomega.4c09986 39959076 PMC11822487

[18] Chen W, Yao J, Meng J, Han W, Tao Y, Chen Y, et al. Promiscuous enzymatic activity-aided multiple-pathway network design for metabolic flux rearrangement in hydroxytyrosol biosynthesis. Nat Commun. 2019;10(1):960. doi: 10.1038/s41467-019-08781-2 30814511 PMC6393456

[19] Yao J, He Y, Su N, Bharath SR, Tao Y, Jin J-M, et al. Developing a highly efficient hydroxytyrosol whole-cell catalyst by de-bottlenecking rate-limiting steps. Nat Commun. 2020;11(1):1515. doi: 10.1038/s41467-020-14918-5 32251291 PMC7090077

[20] Motta P, Molla G, Pollegioni L, Nardini M. Structure-Function Relationships in l-Amino Acid Deaminase, a Flavoprotein Belonging to a Novel Class of Biotechnologically Relevant Enzymes. J Biol Chem. 2016;291(20):10457–75. doi: 10.1074/jbc.M115.703819 27022028 PMC4865898

[21] Wang Y, Bai Y, Fan T, Zheng X, Cai Y. Reducing 3,4-dihydroxyphenylpyruvic acid to D-3,4-dihydroxyphenyllactic acid via a coenzyme nonspecific D-lactate dehydrogenase from Lactobacillus reuteri. J Appl Microbiol. 2019;125:1739–48. doi: 10.1111/jam.14077 30129993

[22] Chen X. Cloning, expression and characterization of a glucose dehydrogenase from Bacillus sp. G3 in Escherichia coli. Afr J Microbiol Res. 2011;5(32). doi: 10.5897/ajmr11.831

[23] Rashid F-ZM, Crémazy FGE, Hofmann A, Forrest D, Grainger DC, Heermann DW, et al. The environmentally-regulated interplay between local three-dimensional chromatin organisation and transcription of proVWX in E. coli. Nat Commun. 2023;14(1):7478. doi: 10.1038/s41467-023-43322-y 37978176 PMC10656529

[24] Peng S, Stephan R, Hummerjohann J, Tasara T. Evaluation of three reference genes of Escherichia coli for mRNA expression level normalization in view of salt and organic acid stress exposure in food. FEMS Microbiol Lett. 2014;355(1):78–82. doi: 10.1111/1574-6968.12447 24766447

[25] Fu SLL, Qiu F, Wang B, Du W, Feng L. Application of a simple and rapid LC-MS/MS method for determination of danshensu in human plasma for an oral pharmacokinetic study of danshen granules in Chinese. Anal Methods-Uk. 2014;6. doi: 10.1039/c3ay42134a

[26] Li J, Pi C, Zhang J, Jiang F, Bao T, Gao L, et al. Fungal bioconversion of lignin-derived aromatics: Pathways, enzymes, and biotechnological potential. Biotechnol Adv. 2025;83:108624. doi: 10.1016/j.biotechadv.2025.108624 40505753

[27] Li R, Zhang R, Xian M, Zou H. Biocatalytic nitration of aromatic and heterocyclic compounds: current status and future prospects. Adv Synth Catal. 2026;368(3). doi: 10.1002/adsc.70279

[28] Li Z, Wang X, Hu G, Li X, Song W, Wei W, et al. Engineering metabolic flux for the microbial synthesis of aromatic compounds. Metab Eng. 2025;88:94–112. doi: 10.1016/j.ymben.2024.12.007 39724940

[29] Huang Z, Li N, Yu S, Zhang W, Zhang T, Zhou J. Systematic Engineering of Escherichia coli for Efficient Production of Nicotinamide Mononucleotide From Nicotinamide. ACS Synth Biol. 2022;11(9):2979–88. doi: 10.1021/acssynbio.2c00100 35977419

[30] Xiong T, Li X, Liu W, Yue H, Liu J, Bai D, et al. Multienzyme cascade for synthesis of hydroxytyrosol via engineered Escherichia coli. Sci Rep. 2025;15(1):471. doi: 10.1038/s41598-024-84624-5 39748076 PMC11696566

[31] Shen X, Zhou D, Lin Y, Wang J, Gao S, Kandavelu P, et al. Structural Insights into Catalytic Versatility of the Flavin-dependent Hydroxylase (HpaB) from Escherichia coli. Sci Rep. 2019;9(1):7087. doi: 10.1038/s41598-019-43577-w 31068633 PMC6506529

[32] Sharma VK, Hutchison JM, Allgeier AM. Redox Biocatalysis: Quantitative Comparisons of Nicotinamide Cofactor Regeneration Methods. ChemSusChem. 2022;15(22):e202200888. doi: 10.1002/cssc.202200888 36129761 PMC10029092

[33] Lee YS, Gerulskis R, Minteer SD. Advances in electrochemical cofactor regeneration: enzymatic and non-enzymatic approaches. Curr Opin Biotechnol. 2022;73:14–21. doi: 10.1016/j.copbio.2021.06.013 34246871

[34] Zhang J-D, Li A-T, Yu H-L, Imanaka T, Xu J-H. Synthesis of optically pure S-sulfoxide by Escherichia coli transformant cells coexpressing the P450 monooxygenase and glucose dehydrogenase genes. J Ind Microbiol Biotechnol. 2011;38(5):633–41. doi: 10.1007/s10295-010-0809-3 20721599

[35] Tan Z, Clomburg JM, Gonzalez R. Synthetic Pathway for the Production of Olivetolic Acid in Escherichia coli. ACS Synth Biol. 2018;7(8):1886–96. doi: 10.1021/acssynbio.8b00075 29976061

[36] Zhou L, Ding Q, Jiang G-Z, Liu Z-N, Wang H-Y, Zhao G-R. Chromosome engineering of Escherichia coli for constitutive production of salvianic acid A. Microb Cell Fact. 2017;16(1):84. doi: 10.1186/s12934-017-0700-2 28511681 PMC5434548

[37] Lu H, Bai Y, Fan T, Zhao Y, Zheng X, Cai Y. Identification of a l-Lactate dehydrogenase with 3,4-dihydroxyphenylpyruvic reduction activity for l-Danshensu production. Process Biochemistry. 2018;72:119–23. doi: 10.1016/j.procbio.2018.06.011

[38] Li C, Zhang Z, Wang J. A thermophilic biofunctional multienzyme cascade reaction for cell-free synthesis of salvianic acid A and 3,4-dihydroxymandelic acid. ACS Sustain Chem Eng. 2019;7(22):18247–53. doi: 10.1021/acssuschemeng.9b05496

[39] Han R, Gao K, Jiang Y, Zhou J, Xu G, Dong J, et al. Self-Sufficient In Vitro Multi-Enzyme Cascade for Efficient Synthesis of Danshensu from l-DOPA. ACS Synth Biol. 2023;12(1):277–86. doi: 10.1021/acssynbio.2c00552 36412006

[40] Yang J, Wei W, Gao C, Song W, Gao C, Chen X, et al. Efficient production of salvianic acid A from L-dihydroxyphenylalanine through a tri-enzyme cascade. Bioresour Bioprocess. 2023;10(1):31. doi: 10.1186/s40643-023-00649-0 38647923 PMC10992476

[41] Tian D, Tian G, Qin Z, Wang S, Wu S, Zhang P, et al. Engineering of Escherichia coli cytoplasm and periplasm for efficient synthesis of salvianic acid A. Metab Eng. 2025;91:415–29. doi: 10.1016/j.ymben.2025.06.006 40532757

[42] Qi S, Zhai X, Chen Z, Wang L, Ji L, Zhuang H, et al. Chemo-enzymatic cascade synthesis of d -danshensu and its derivatives from lignin. Green Chem. 2026;28(23):9911–23. doi: 10.1039/d6gc01908k

