# Supplementary material for: Multienzyme cascade biotransformation of L-Tyrosine to Danshensu by engineered Escherichia coli
Source: PLoS One. 2026 Oct 23;21(10):e0361484. doi: 10.1371/journal.pone.0361484 (PMC13693876; doi:10.1371/journal.pone.0361484)
Supplement: Supplementary file 1 [file pone.0361484.s001.docx]

**SUPPLEMENTARY MATERIAL**

**Materials and methods**

**Construction of plasmids and strains**

The *HpaBC* was amplified using primers of *HpaBC*-*Eco*RI (F) and *HpaBC*-*Sac*I (R) with the genome sequence of *E. coli* BL21(DE3) as template. The amplified fragment was cloned into the pRSFDuet-1 and pETDuet-1 between the *Eco*RI and *Sac*I. The constructed plasmids were given the names pRSF-*HpaBC* and pET-*HpaBC*. The *aadL* was amplified using primers of *aadL*-*Eco*RI (F) and *aadL*-*Sac*I (R). The amplified fragment was cloned into the pRSFDuet-1, pETDuet-1 between the *Eco*RI and *Sac*I. The constructed plasmids were given the names pRSF-*aadL* and pET-*aadL*. The *ldhD* was amplified using primers of *ldhD*-*Eco*RI (F) and *ldhD*-*Sac*I (R). The amplified fragment was cloned into the pETDuet-1 between the *Eco*RI and *Sac*I. The constructed plasmids were given the names pET-*ldhD*. The *gdh* was amplified using primers of *gdh*-*Eco*RI (F) and *gdh*-*Sac*I (R). The amplified fragment was cloned into pETDuet-1, pCDFuet-1 between the *Eco*RI and *Sac*I. The constructed plasmids were given the names pET-*gdh* and pCDF-*gdh*.

The *aadL* was amplified using primers of *aadL*-*Kpn*I (F) and *aadL*-*Xho*I (R). The amplified fragment was cloned into pRSF-*HpaBC* between the *Kpn*I and *Xho*I. The constructed plasmids were given the names pRSF-*HpaBC*-*aadL*. The *gdh* was amplified using primers of *gdh*-*Kpn*I (F) and *gdh*-*Xho*I (R). The amplified fragment was cloned into pRSF-*HpaBC*, pRSF-*aadL*, pET-*HpaBC* and pET-*aadL* between *Kpn*I and *Xho*I. The constructed plasmids were given the names pRSF-*HpaBC*-*gdh*, pRSF-*aadL*-*gdh*, pET-*HpaBC*-*gdh* and pET-*aadL*-*gdh*, respectively. The *ldhD* was amplified using primers of *ldhD*-*Kpn*I (F) and *ldhD*-*Xho*I (R). The amplified fragment was cloned into pRSF-*HpaBC*, pRSF-*aadL*, pET-*gdh*, pET-*HpaBC*, pET-*aadL* and pCDF-*gdh* between *Kpn*I and *Xho*I. The constructed plasmids were given the names pRSF-*HpaBC*-*ldhD*, pRSF-*aadL*-*ldhD*, pET-*gdh*-*ldhD*, pET-*HpaBC*-*ldhD*, pET-*aadL*-*ldhD* and pCDF-*gdh*-*ldhD*, respectively.

We transferred the constructed plasmids into *E. coli* BL21 (DE3). The constructed strains were given the names: strain 1 (pRSF-*HpaBC*-*aadL*/pET-*gdh*-*ldhD*), strain 2 (pRSF-*aadL*-*gdh*/pET-*HpaBC*-*ldhD*), strain 3 (pRSF-*HpaBC*-*gdh*/pET-*aadL*-*ldhD*), strain 4 (pRSF-*aadL*-*ldhD*/pET-*HpaBC*-*gdh*), strain 5 (pRSF-*HpaBC*-*ldhD*/pET-*aadL*-*gdh*), strain 6 (pRSF-*HpaBC*-*aadL*/pCDF-*gdh*-*ldhD*), and strain 7 (pRSF-*HpaBC*-*aadL*/pET-*ldhD*/pCDF-*gdh*).

**Analytical Methods.**

Ultraperformance liquid chromatography−mass spectrometry (UPLC−MS, Waters Acquity UPLC and Waters MALDI SYNAPT Q-TOF MS, Milford, MA, U.S.A.) was used to confirm the formation of Danshensu (DSS).

**Supporting Figure**

**Fig. S1** UPLC−MS assay of DSS, (A) mass spectrometry of DSS, (C) UV absorption spectra of DSS

**Supporting Tables**

**Table S1:** Primers used in the work

| Primers | Sequences, 5’-3’ |
| --- | --- |
| *HpaBC-Eco*RI (F) | GCCG**GAATTC**AAACCAGAAGATTTCCGCGCC |
| *HpaBC-Sac*I (R) | GCCG**GAGCTC**TTAAATCGCAGCTTCCATTTC |
| *aadL-Eco*RI (F) | GCCG**GAATTC**ATGAATATTTCTCGCCGTAAAC |
| *aadL-Sac*I (R) | GCCG**GAGCTC**TTACTTCTTGAAACGGTCAAG |
| *aadL-**Kpn*I (F) | GCCG**CATATG**ATGAATATTTCTCGCCGTAAAC |
| *aadL-Xho*I (R) | GCCG**CTCGAG**TTACTTCTTGAAACGGTCAAG |
| *ldhD-Kpn*I (F) | GCCG**CATATG**ATGACGAAAATCTTGATGAC |
| *ldhD-Xho*I (R) | GCCG**CTCGAG**TTATAAGGTGATTTCATGTGG |
| *gdh-Eco*RI (F) | GCCG**GAATTC**ATGTACTCGGACCTGGAAGG |
| *gdh-Sac*I (R) | GCCG**GAGCTC**TTAACCGCGACCTGCCTGAAATG |
| *gdh-Kpn*I (F) | GCCG**CATATG**ATGTACTCGGACCTGGAAGG |
| *gdh-Xho*I (R) | GCCG**CTCGAG**TTAACCGCGACCTGCCTGAAATG |
| RT-*HpaBC* (F) | CGTCATTTGCCGACCGATAA |
| RT-*HpaBC* (R) | AGTTGGTGGCAACCACTTTC |
| RT-*aadL* (F) | GTGGCGTGGTATGAACGAAA |
| RT-*aadL* (R) | AGCCGTTTCTTTGGCTGTTT |
| RT-*ldhD* (F) | GCGATGAACAGTCAACCGAA |
| RT-*ldhD* (R) | GCACCGAGGGCATTAAGAAG |
| RT-*gdh* (F) | ACTCGGACCTGGAAGGAAAG |
| RT-*gdh* (R) | TCGCGAGAGCGGTAGTTAAT |
| RT-*16S* (F) | CTCTTGCCATCGGATGTGCCCA |
| RT-*16S* (R) | CCAGTGTGGCTGGTCATCCTCTCA |

**Table S2：**Strains and plasmids used in this study

| Strains and plasmids | Description | Source |
| --- | --- | --- |
| pRSFDuet-1 | Double T7 promoters, RSF ori, Kan^R^ | Novagen |
| pETDuet-1 | Double T7 promoters, pBR322 ori, Amp^R^ | Novagen |
| pCDFDuet-1 | Double T7 promoters, CDF13 ori, Sm^R^ | Novagen |
| pRSF-*HpaBC*-*aadL* | pRSFDuet-1 carrying *HpaBC* and *aadL* | This study |
| pRSF-*HpaBC*-*gdh* | pRSFDuet-1 carrying *HpaBC* and *gdh* | This study |
| pRSF-*HpaBC*-*ldhD* | pRSFDuet-1 carrying *HpaBC* and *ldhD* | This study |
| pRSF-*aadL*-*ldhD* | pRSFDuet-1 carrying *aadL* and *ldhD* | This study |
| pRSF-*aadL*-*gdh* | pRSFDuet-1 carrying *aadL* and *gdh* | This study |
| pET-*gdh*-*ldhD* | pETDuet-1 carrying *gdh* and *ldhD* | This study |
| pET-*HpaBC*-*ldhD* | pETDuet-1 carrying *HpaBC* and *ldhD* | This study |
| pET-*aadL*-*ldhD* | pETDuet-1 carrying *aadL* and *ldhD* | This study |
| pET-*HpaBC*-*gdh* | pETDuet-1 carrying *HpaBC* and *gdh* | This study |
| pET-*aadL*-*gdh* | pETDuet-1 carrying *aadL* and *gdh* | This study |
| pET-*ldhD* | pETDuet-1 carrying *ldhD* | This study |
| pCDF-*gdh*-*ldhD* | pCDFDuet-1 carrying *gdh* and *ldhD* | This study |
| pCDF-*gdh* | pCDFDuet-1 carrying *gdh* | This study |
| Strain 1 | *E.coli* BL21(DE3) harboring pRSF-*HpaBC*-*aadL* and pET-*gdh*-*ldhD* | This study |
| Strain 2 | *E.coli* BL21(DE3) harboring pRSF-*aadL*-*gdh* and pET-*HpaBC*-*ldhD* | This study |
| Strain 3 | *E.coli* BL21(DE3) harboring pRSF-*HpaBC*-*gdh* and pET-*aadL*-*ldhD* | This study |
| Strain 4 | *E.coli* BL21(DE3) harboring pRSF-*aadL*-*ldhD* and pET-*HpaBC*-*gdh* | This study |
| Strain 5 | *E.coli* BL21(DE3) harboring pRSF-*HpaBC*-*ldhD* and pET-*aadL*-*gdh* | This study |
| Strain 6 | *E.coli* BL21(DE3) harboring pRSF-*HpaBC*-*aadL* and pCDF-*gdh*-*ldhD* | This study |
| Strain 7 | *E.coli* BL21(DE3) harboring pRSF-*HpaBC*-*aadL*, pET-*ldhD* and pCDF-*gdh* | This study |
